# Novel approach to hydroxy-group-containing porous organic polymers from bisphenol A

**DOI:** 10.3762/bjoc.13.211

**Published:** 2017-10-12

**Authors:** Tao Wang, Yan-Chao Zhao, Li-Min Zhang, Yi Cui, Chang-Shan Zhang, Bao-Hang Han

**Affiliations:** 1 CAS Key Laboratory of Nanosystem and Hierarchical Fabrication, CAS Center for Excellence in Nanoscience, National Center for Nanoscience and Technology, Beijing 100190, China; 2 School of Chemical Engineering, Nanjing University of Science and Technology, Nanjing 210094, China

**Keywords:** bisphenol A, carbon dioxide uptake, hydrogen storage, OH-containing, porous organic polymers

## Abstract

We successfully employed bisphenol A and several different formyl-containing monomers as useful building blocks to construct a series of hydroxy-group-containing porous organic polymers in a sealed tube at high temperature. Fourier transform infrared and solid-state ^13^C CP/MAS NMR spectroscopy are utilized to characterize the possible structure of the obtained polymers. The highest Brunauer–Emmet–Teller specific surface area of the phenolic-resin porous organic polymers (PPOPs) is estimated to be 920 m^2^ g^–1^. The PPOPs exhibit a highest carbon dioxide uptake (up to 15.0 wt % (273 K) and 8.8 wt % (298 K) at 1.0 bar), and possess moderate hydrogen storage capacities ranging from 1.28 to 1.04 wt % (77 K) at 1.0 bar. Moreover, the highest uptake of methane for the PPOPs is measured as 4.3 wt % (273 K) at 1.0 bar.

## Introduction

Porous organic polymers standing out from kinds of porous materials such as zeolite, activated carbon, metal-organic frameworks [1–2], and covalent organic frameworks [3–4], with their prominent potential as heterogeneous catalysts [5–7], supports for catalysts [8–9], gas permeable membranes [10–11], and gas storage materials [12–14] have attracted much attentions from researchers all over the world as reviewed by Matyjaszewski [15]. During the past years, a large amount of porous organic polymers (POPs) have been reported via Sonogashira–Hagihara coupling reaction [16], Suzuki–Miyaura chemistry [17], Yamato reaction [18], and self condensation of aromatic nitriles[19]. Although these methods can be used to construct POPs with high specific surface area values, these reactions are usually catalyzed by heavy and/or transition-metal catalysts, which are usually expensive and environmentally harmful. Furthermore, the majority of reagents used for the preparation of the aforementioned POPs are synthesized through multiple steps at high cost. New reactions using inexpensive and convenient raw materials with non-metallic catalysts, even no catalysts might show great advantages for construction of porous organic polymers. Our group has made much contribution to the exploitation of such reaction methodologies without any metallic catalysts [20–22].

We found that Bakelite-type chemistry is a reaction that can be catalyzed without any metal-containing catalysts and it is selected as an appropriate approach, spontaneously. Phenolic resins can be produced commercially using bases (ammonia and sodium hydroxide) or acids (hydrochloric acid and sulfuric acid) as catalysts via connecting phenolic molecules with formaldehyde or other aromatic aldehydes to form cross-linking structures through the simple Bakelite-type chemistry to obtain great number of polymers with different functionalities. Phloroglucinol has been utilized as a monomer by Kanatzidis and Katsoulidis [23–24] to produce a series of porous polymers. In this contribution, bisphenol A (BPA) was employed as a novel polyphenol monomer instead of phloroglucinol. BPA is a commercially available industrial raw material, which is much cheaper than phloroglucinol. In addition, BPA might be stored more easily, compared with phloroglucinol that has a relatively high reactivity and can be oxidized in an ambient environment. Based on the aforementioned, BPA, to the best of our knowledge, may be a suitable candidate prior to other phenolic compounds such as phloroglucinol [23] and 1,5-dihydroxynaphthalene [24], which have been used for the preparation of porous materials.

Recently, Kanatzidis and Katsoulidis have reported a series of Bakelite-type porous organic polymers prepared in two steps. The mixture of reagents and solvent was pretreated at 70 °C and kept for 1 h, followed by a high temperature treatment at 220 °C for 96 h [25]. This approach is involved with a longer reaction time and a higher reaction temperature, which might cause a tremendous energy waste. Herein we provide an effective one-step approach to construct phenolic-resin porous organic polymers (PPOPs) from the reactions between BPA and different aldehydes using *p*-toluenesulfonic acid (TSA) as catalyst that has been proved to be a non-metallic acidic catalyst with high efficiency [26–27]. The materials exhibit Brunauer–Emmet–Teller (BET) specific surface area values ranging from 720 to 920 m^2^ g^–1^, and the highest carbon dioxide uptake is up to 15.0 wt % at 273 K and 1.0 bar. Meanwhile, the hydrogen and methane capacities are also investigated. Considering the gas adsorption properties, PPOPs may be a promising candidate for gas storage and separation materials.

## Results and Discussion

**Scheme 1:**
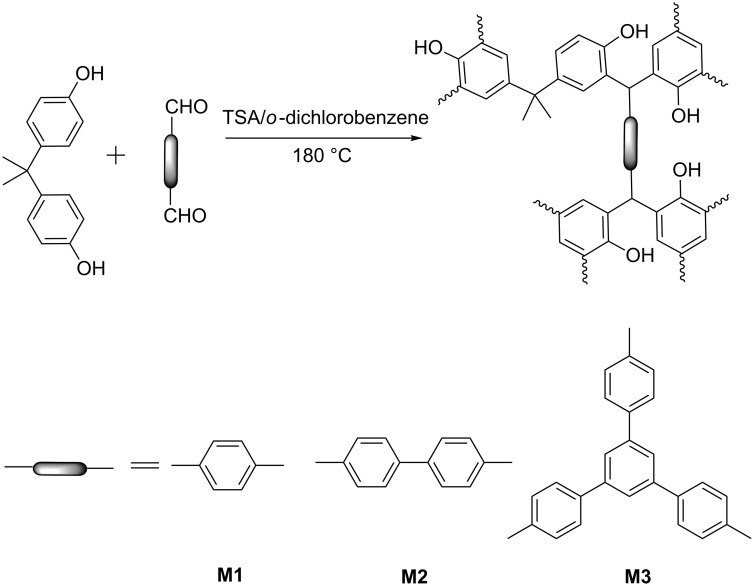
Schematic representation of the possible structures of bisphenol-A-based porous organic polymers.

Three multi-formyl compounds, i.e., two dialdehydes **M1** and **M2** [22] and one trialdehyde **M3** [22] were employed to react with bisphenol A to produce phenolic-resin porous polymers **PPOP-1**–**PPOP-3** (Scheme 1). It is well-known that the *ortho*- and *para*-position of phenol are activated with negative charge for the electrophilic aromatic substitution in consequence of the electron-donating effect of the hydroxy group. The positively charged carbonyl group of the aldehyde could be attacked by the electron-rich phenyl ring, thus a carbonyl group can connect two phenol molecules by elimination of a water molecule. As a result, a cross-linked hydroxy-group-containing polymer is constructed ultimately. BPA and multi-formyl-containing compounds are suspended in *o*-dichlorobenzene, and TSA, as a catalyst, was then added into reaction system. After the reaction in a sealed tube at 180 °C for 72 h, three polymers **PPOP-1**–**PPOP-3** were obtained. The possible chemical structures of the obtained PPOPs are shown in the Scheme 1. All of the polymers are stable and insoluble in common organic solvents such as dichloromethane, ethanol, and acetone. Furthermore, the as-prepared materials exhibit a high thermal stability according to the results of TGA (Supporting Information File 1, Figure S1). There is a weight loss of about 5% up to 150 °C, which is attributed to the evaporation of trapped solvent, carbon dioxide, or adsorbed water that could not be easily removed from the microporous structure of the polymers during the after-synthesis treatment and drying process. There is not any obvious thermal degradation for PPOPs until when heated up to 300 °C.

**Figure 1:**
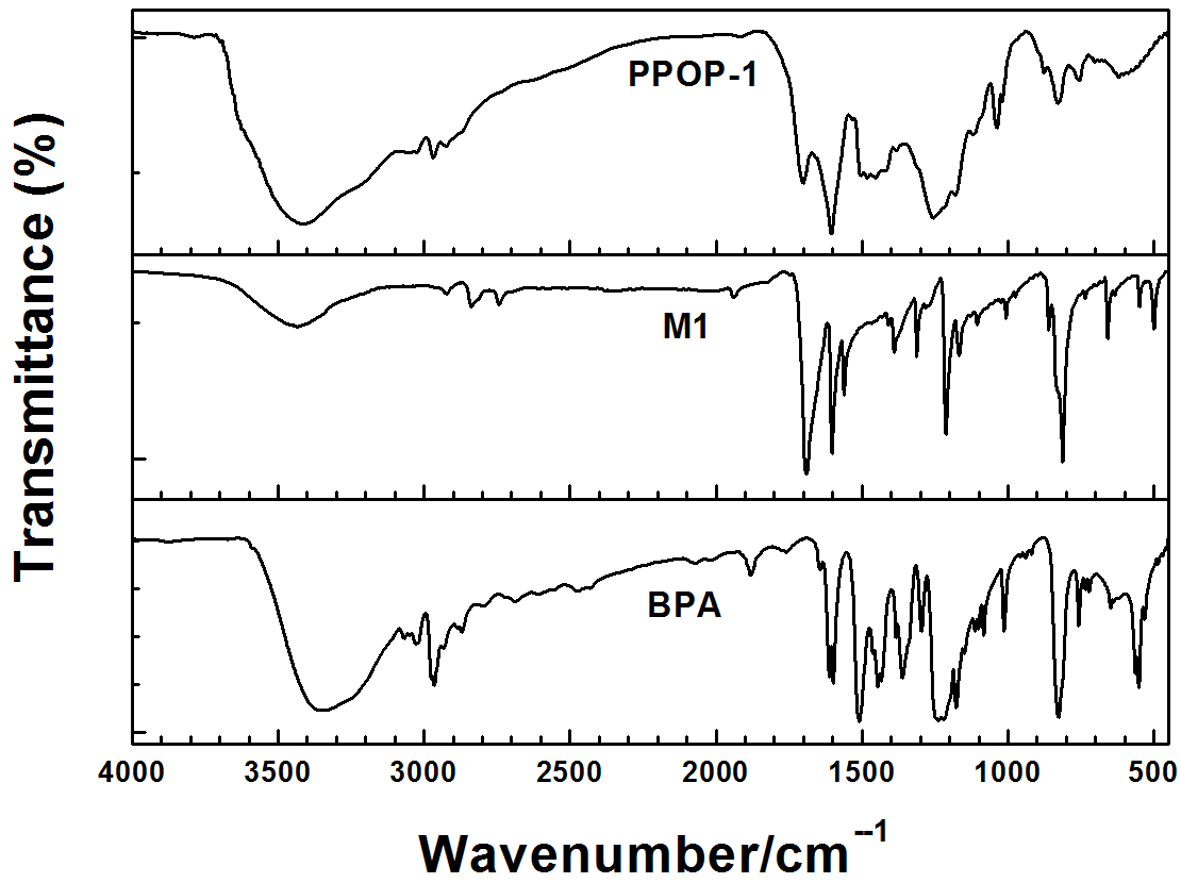
FTIR spectra of terephthalic aldehyde (**M1**), BPA, and **PPOP-1**.

According to the TGA result of BPA (Supporting Information File 1, Figure S2), the thermal degradation of BPA begins around 180 °C and the evolved products are mainly phenol with one or two benzene rings from investigations of thermal degradation of bisphenol A polycarbonate [28]. Investigation of the changes from methyl groups and aldehyde groups between monomers and polymers is carried out by means of FTIR spectroscopy. The FTIR spectra displayed (Figure 1 and Supporting Information File 1, Figure S3) that signal at 2970 cm^−1^ arised from the stretching vibration of methyl groups shows quantitative changes between BPA and PPOPs, suggesting the degradation of BPA in *o*-dichlorobenzene and it is reported that the cleavage of methylene can be catalyzed under acidic or basic conditions [29–30]. *p*-Toluenesulfonic acid acted as an acid catalyst in this contribution and will promote the cleavage of BPA at high temperature. The broad absorption bands located at ca. 3500 cm^−1^ is attributed to the characteristic stretching vibration of hydroxy groups, which is consistent with the literature data [25]. The absorption peak at 1705 cm^−1^ assigned to the stretching vibration of carbonyl groups is significantly reduced in PPOPs, indicating that most of the aldehyde compounds are consumed.

**Figure 2:**
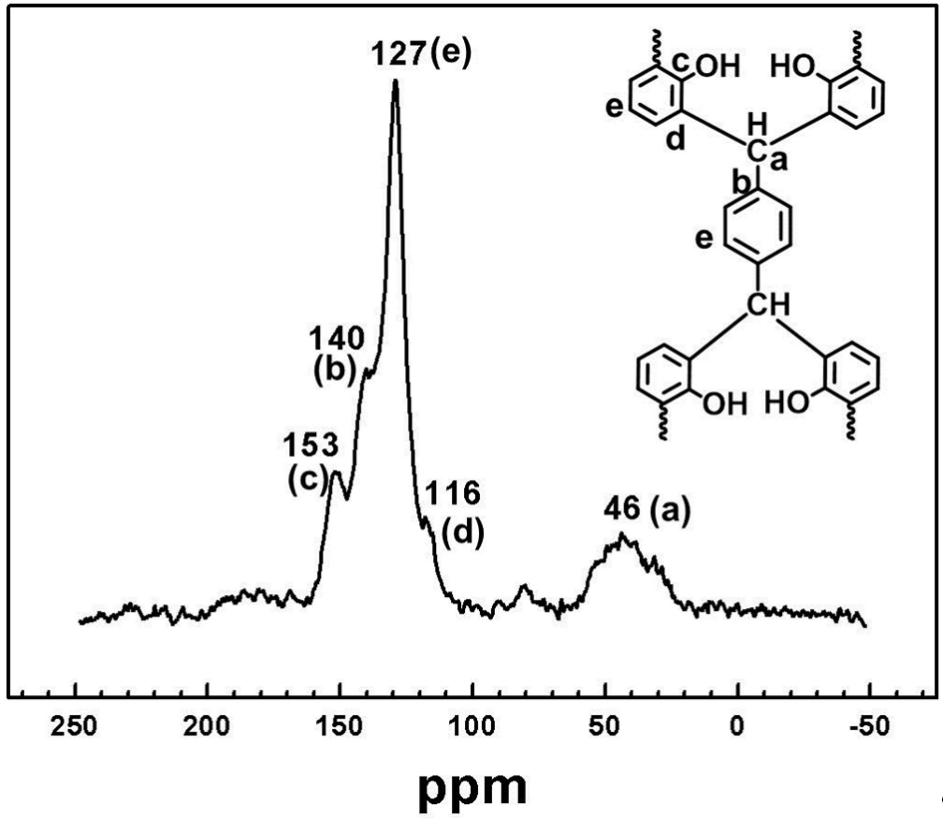
Solid-state ^13^C CP/MAS NMR spectrum of **PPOP-1** recorded at the MAS rate of 5 kHz.

Solid-state ^13^C CP/MAS NMR spectroscopy was employed to characterize the structure of the polymers PPOPs. As shown in Figure 2 and Figure S4 (Supporting Information File 1), it can be found that the ^13^C chemical shifts of these polymers are similar. It is reasonable considering the structural features of these polymers. Typically, taking the spectrum of **PPOP-1** for example, two major resonances at 127 and 140 ppm are assigned to the unsubstituted phenyl carbon atoms and the substituted aromatic carbon atoms, respectively. The resonance at 46 ppm can be ascribed to the tertiary carbon atoms that act as the linkage of two different benzene rings originated from the BPA and aldehyde monomers, respectively. The shoulder peak at 116 ppm is related to the reacted *ortho* carbons of the hydroxy groups. The signal at 153 ppm comes from the phenoxy carbons [25]. Unexpectedly, no obvious peak at 20 ppm ascribed to the carbons of methyl groups from BPA molecules can be found in the spectrum of **PPOP-1**, which is in consistence with the results of FT-IR spectra, indicating the degradation of BPA catalyzed by TSA at high temperature [29–30].

**Figure 3:**
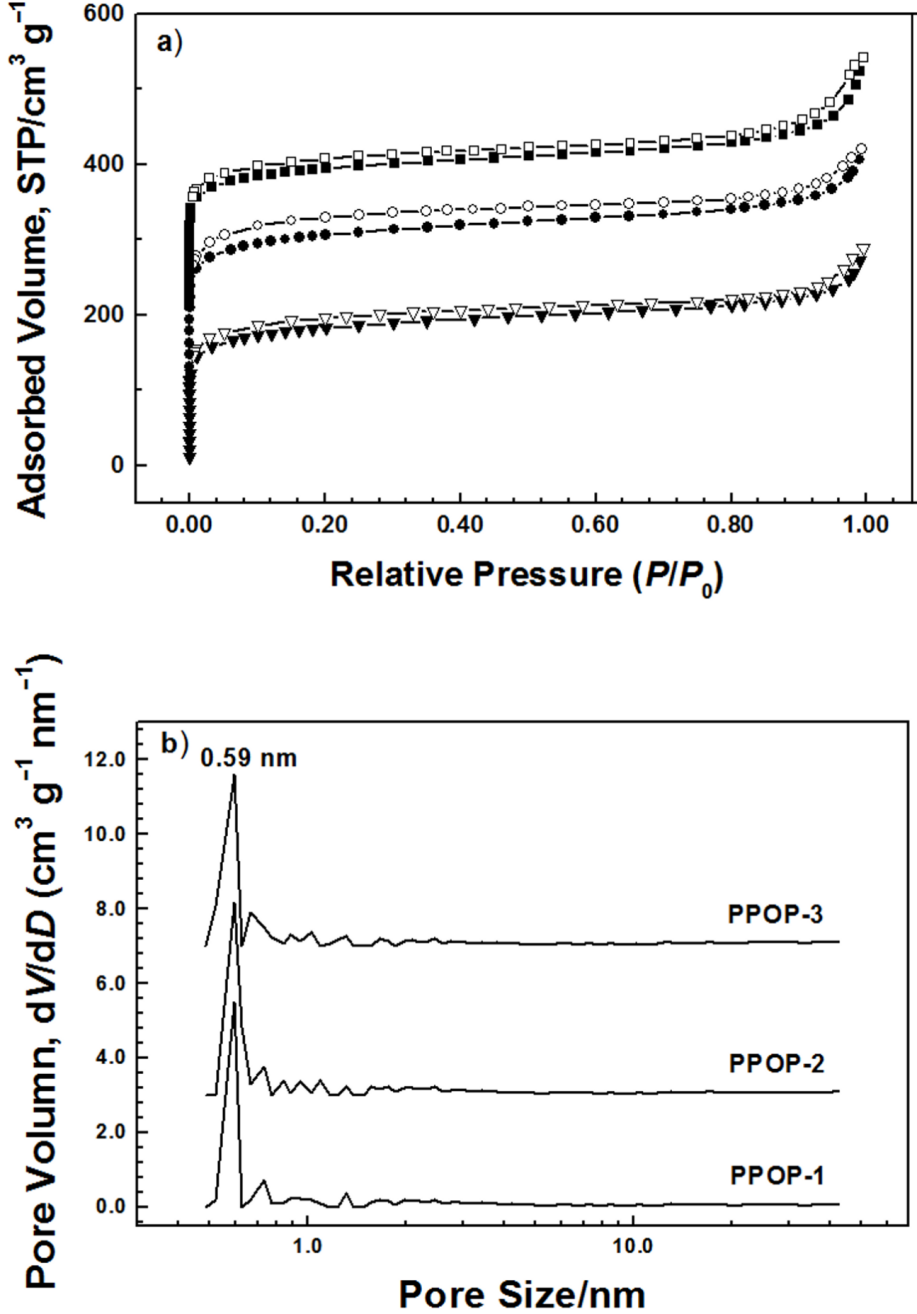
(a) Nitrogen adsorption–desorption isotherms of **PPOP-1** (downtriangle), **PPOP-2** (circle), and **PPOP-3** (square) at 77 K. The isotherms have been offset by 100 cm^3^ g^−1^ for **PPOP-2** and 200 cm^3^ g^−1^ for **PPOP-3** for the purpose of clarity, respectively. (b) PSD profiles calculated by the original DFT method. The PSD profiles of **PPOP-2** and **PPOP-3** have been offset by 3 and 6 units for the purpose of clarity, respectively.

Nitrogen sorption measurements were employed to evaluate the porosity of the obtained polymers. The nitrogen adsorption–desorption isotherms of **PPOP-1**–**PPOP-3** are similar to each other (Figure 3a). All of the isotherms show a high gas uptake at relative pressure (*P*/*P*_0_) less than 0.02, indicating that the materials are microporous. Meanwhile, a nitrogen condensation step could be found for all the polymers at *P*/*P*_0_ above 0.90, which is an indication of characteristic macroporosity that might correspond to interparticular voids associated with the pack of small particles of about 4 μm adhered to the external surface of spherical particles (Supporting Information File 1, Figure S5). The BET specific surface area values are calculated in the relative pressure range *P*/*P*_0_ = 0.01–0.10 for the microporous materials [31] for PPOPs (Supporting Information File 1, Figure S6). **PPOP-2** possesses the highest BET surface area value calculated as 920 m^2^ g^–1^. According to the obtained values summarized in Table 1, both total pore volume (0.36 cm^3^ g^–1^) determined at *P*/*P*_0_ = 0.95 and micropore volume (0.18 cm^3^ g^–1^) calculated using the *t*-plot method of **PPOP-1** are smaller than those of **PPOP-2** and **PPOP-3**. The difference between the pore volumes and BET specific surface area results of PPOPs may be related to the monomer strut length. With the shortest linker of **M1**, **PPOP-1** possesses the lowest pore volume and BET specific surface area. As for **PPOP-3**, using **M3** as a monomer may induce a depression of polymerization degree owing to its stereo-hindrance effect, which might be responsible for its lower BET surface area value (880 m^2^ g^−1^) and micropore volume (0.20 cm^3^ g^−1^) than that of **PPOP-2** using **M2** as the monomer. However, it is noteworthy that when the reaction is conducted between **M1** and phenol selected as the substitution of BPA, a new material is obtained with a BET surface area value calculated as 470 m^2^ g^−1^ (Supporting Information File 1, Figure S7), which is a indication of the fact that pyrolysis of BPA might result in some new porous structure in situ, leading to an increase in BET surface area value. The PSD profiles calculated using original DFT are shown in Figure 3b. All of the materials exhibit a similar PSD profile with a maximum peak at 0.59 nm and several smaller peaks between 0.6 and 2.0 nm, indicating that PPOPs are microporous. The pore size for PPOPs and the total pore volume for **PPOP-2** and **PPOP-3** do not show any obvious difference with increasing monomer strut length, which may be attributed to the random penetration and space-filling within the fragments of the extended repeating units [16].

**Table 1:** Porosity properties of **PPOP-1**–**PPOP-3**.

polymer	*S*_BET_ (m^2^ g^−1^)^a^	*V*_total_ (cm^3^ g^−1^)^b^	*V*_micro_ (cm^3^ g^−1^)^c^

**PPOP-1** **PPOP-2** **PPOP-3**	720 920 880	0.36 0.41 0.41	0.18 0.21 0.20

^a^Surface area calculated from the nitrogen adsorption isotherm using the BET method in the relative pressure (*P*/*P*_0_) range from 0.01 to 0.10. ^b^Total pore volume at *P*/*P*_0_ = 0.95. ^c^Micropore volume calculated from nitrogen adsorption isotherm using the *t*-plot method.

**Figure 4:**
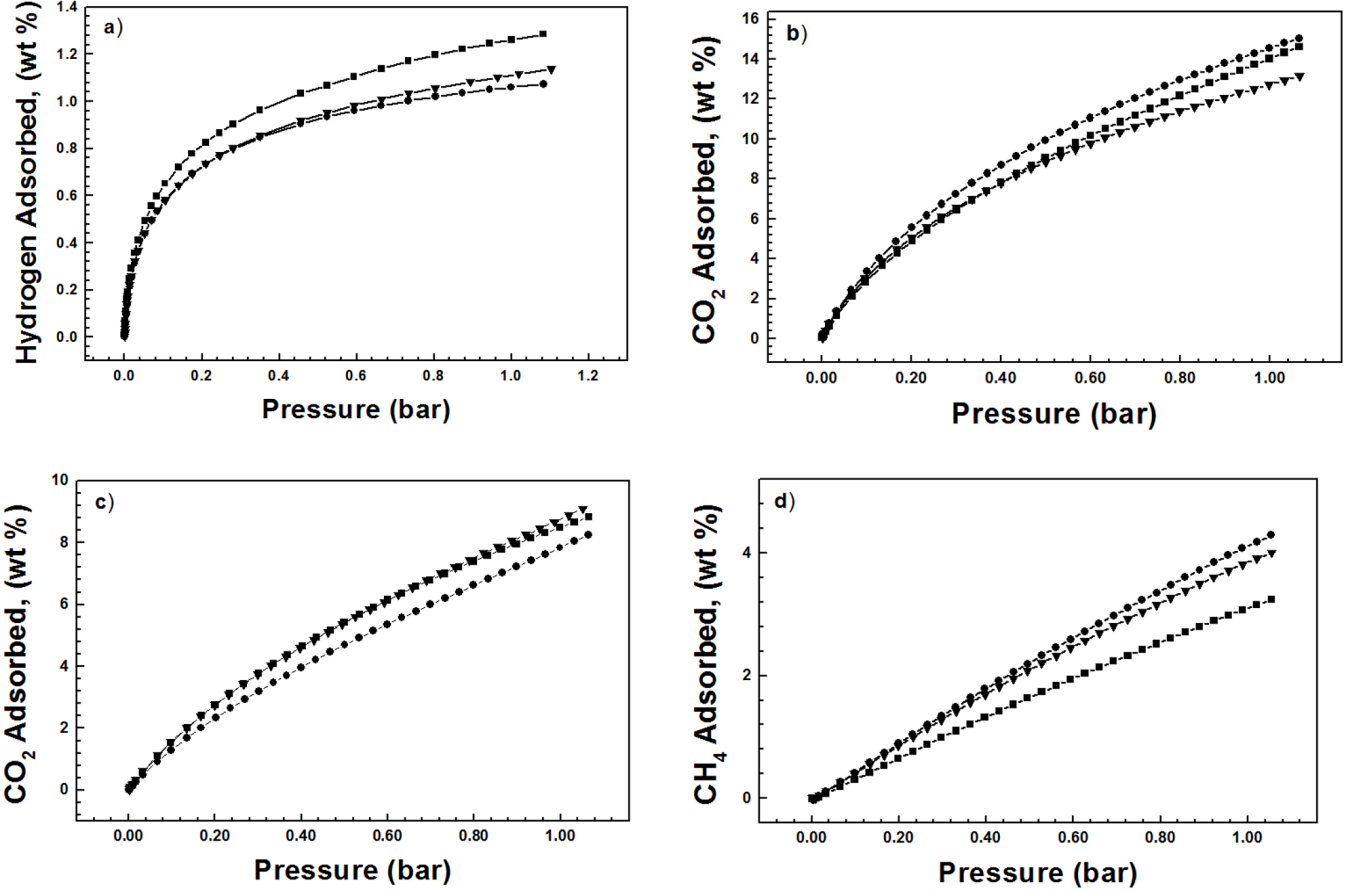
Gravimetric gas adsorption isotherms for **PPOP-1** (downtriangle), **PPOP-2** (circle), and **PPOP-3** (square) (a) hydrogen at 77 K, (b) carbon dioxide at 273 K, (c) carbon dioxide at 298 K, and (d) methane at 273 K.

**Figure 5:**
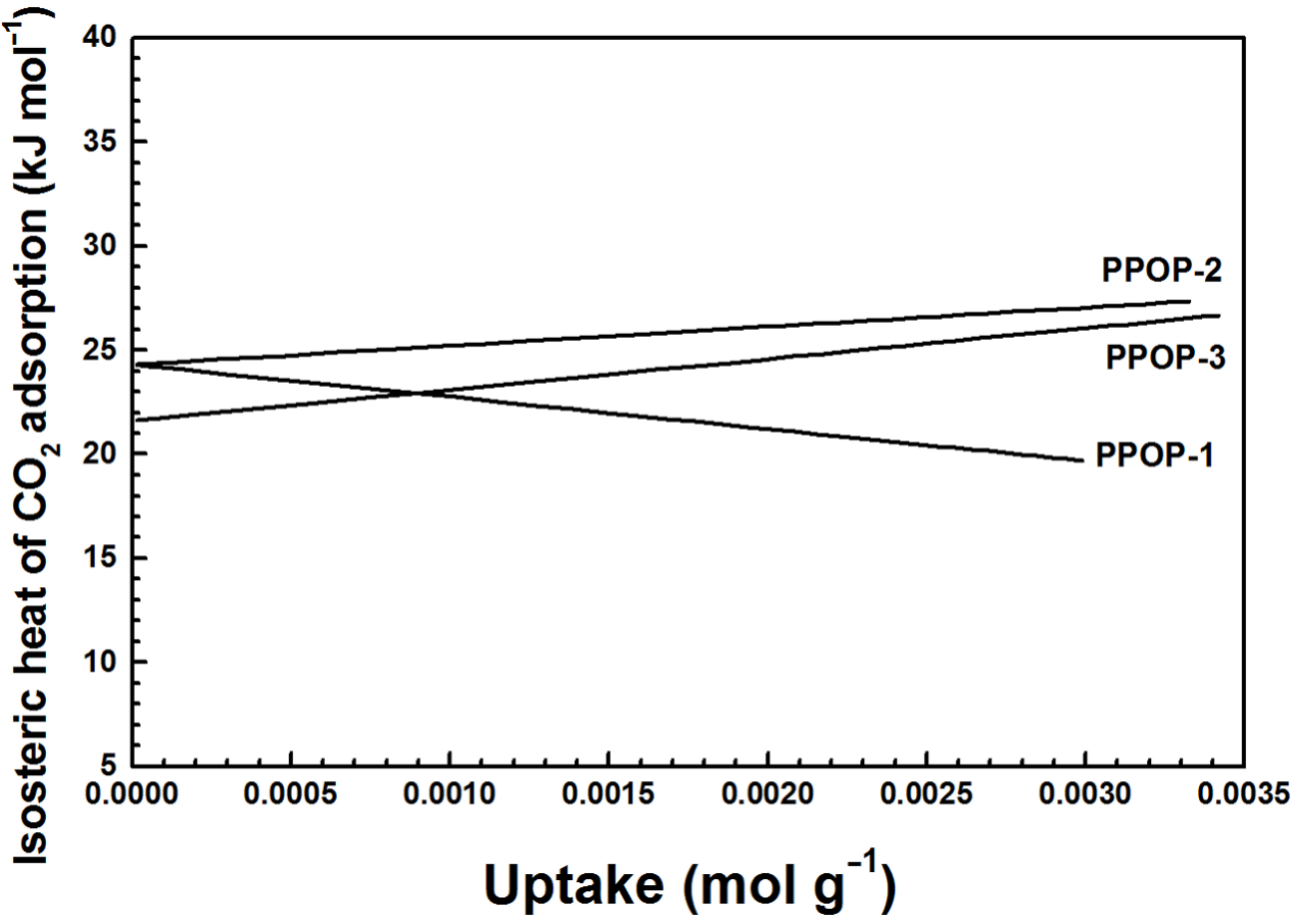
Variation of isosteric heat of adsorption with amount of adsorbed CO_2_ in **PPOP-1**, **PPOP-2**, and **PPOP-3**.

The gas uptake capacities for carbon dioxide, hydrogen, and methane of the polymers are investigated by gravimetric methods and listed in Table 2. The hydrogen storage capacities for PPOPs vary between 1.08 and 1.28 wt % at 77 K and 1.0 bar (Figure 4a) and **PPOP-3** possesses the highest hydrogen uptake, which may be on account of the fact that there is much more ultramicropores in **PPOP-3** that are appropriate for hydrogen rather than nitrogen [32]. The methane gravimetric uptake for the materials was measured at 273 K and 1.0 bar. PPOPs exhibit a methane storage capacity varying between 4.29 and 3.24 wt % (Figure 4d), which is higher than that of the reported mesoporous polymeric organic frameworks (mesoPOF)s [23]. **PPOP-2** with the largest BET surface area and micropore volume shows the highest methane uptake. However, **PPOP-3** possesses a smaller methane storage capacity than **PPOP-1** that is known for its lowest BET surface area and total pore volume and micropore volume, which may arise from the fact that interactions between the accessible surface area, micropore volume, and pore topology contribute predominantly to methane storage capacity in porous material [33–34]. The carbon dioxide adsorption isotherms for PPOPs are collected at 273 and 298 K, respectively (Figure 4b,c). **PPOP-3** possesses a largest carbon dioxide adsorption capacity up to 15.0 wt % (273 K) and 8.8 wt % (298 K) at 1.0 bar, which is larger than that of the reported work [35]. As reported that apparent surface area is not the only crucial factor that influences the amount of adsorbed CO_2_, whereas the uptake capacity is more depended on porosity characteristic such as pore size in the networks [36–37]. Specially, the smallest pores contribute most to the CO_2_ uptake at low pressure [36]. Hence, **PPOP-3** with a smaller pore size located at 0.68 nm that is different from the other two polymers in Figure 3b is probably the best candidate for CO_2_ capture. The high carbon dioxide uptake capacity for PPOPs may correspond to the large amount of the hydroxy groups in the PPOPs through the formation of O=C=O(δ^–^)…H(δ^+^)–O hydrogen bonds that are enhanced by weak supramolecular interactions with C–H atoms on the aromatic rings of the polymers [38]. The isosteric heat of adsorption for carbon dioxide is calculated from adsorption data collected at 273 and 298 K using a virial method and the Clausius–Clapeyron equation [39] (Supporting Information File 1, Figure S8). The typical heats of absorption *Q*_st_ for the PPOPs are measured in the range of about 21.6–24.3 kJ mol^−1^ (Figure 5), which are in accordance with the report data [40], indicating that the adsorption of CO_2_ is mainly physical adsorption. Unusually, **PPOP-2** and **PPOP-3** show an increase in the *Q*_st_ value with increased CO_2_ loading, which is likely induced by synergic interactions between carbon dioxide molecules [41–42].

**Table 2:** Gas adsorption uptake of **PPOP-1**–**PPOP-3**.

polymer	H_2_ uptake (wt %)^a^	CH_4_ uptake (wt %)^b^	CO_2_ uptake (wt %)^c^	

			273 K	298 K

**PPOP-1** **PPOP-2** **PPOP-3**	1.14 1.08 1.28	4.01 4.29 3.24	13.2 14.6 15.0	9.1 8.2 8.8

^a^Hydrogen gravimetric uptake capacities at 77 K measured at hydrogen equilibrium pressure of 1.0 bar. ^b^Methane gravimetric uptake capacities at 273 K measured at a pressure at 1.0 bar. ^c^Carbon dioxide gravimetric uptake capacities at 1.0 bar measured at 273 and 298 K, respectively.

## Conclusion

In conclusion, we have developed a novel approach to porous organic polymers from BPA using the traditional Bakelite-type chemistry. The prepared polymers possess high specific surface area values up to 920 m^2^ g^−1^, with a high carbon dioxide uptake of up to 15.0 wt % at 273 K and 1.0 bar. The materials also exhibit hydrogen uptake properties measured as 1.28 wt % (77 K) at 1.0 bar while the highest methane storage capacity is 4.29 wt % (273 K) at 1.0 bar. These gas adsorption properties and high BET specific surface area may make the PPOPs appropriate candidates for materials for gas adsorption and storage.

## Experimental

### Preparation of PPOPs

A mixture of BPA (50.0 mg, 0.22 mmol), terephthalaldehyde (59.0 mg, 0.44 mmol), and *p*-toluenesulfonic acid (0.5 g) was suspended in *o*-dichlorobenzene (8.0 mL) in a glass tube. After ultrasonication for 0.5 h, the mixture was degassed by at least three freeze–pump–thaw cycles. The tube was frozen at 77 K (liquid nitrogen bath) and evacuated to high vacuum and flame-sealed. After 180 °C for 72 h, the reaction mixture gave a solid product (denoted as **PPOP-1**). After cooled to room temperature, the solid was filtrated and washed with acetone, dichloromethane, and ethanol, subsequently. Further purification of the polymer was carried out by Soxhlet extraction with water, ethanol, and dichloromethane for 24 h to give the final product with a yield of 87.5%, which was dried in vacuo at 120 °C for more than 12 h.

Similar to the preparation of **PPOP-1**, 4,4'-biphenyldicarboxaldehyde (**M2**) and 1,3,5-tri(4-formylphenyl)benzene (**M3**) were used to afford **PPOP-2** and **PPOP-3**, with yield of 85% and 80%, respectively.

## Supporting Information

File 1 Experimental, instruments section, SEM images, data of TGA, FTIR and BET surface area, virial analysis of the adsorption data for CO_2_ and NMR spectra.
