# Supplementary material for: Novel approach to hydroxy-group-containing porous organic polymers from bisphenol A
Source: Beilstein J Org Chem. 2017 Oct 12;13:2131–7. doi: 10.3762/bjoc.13.211 (PMC5647697; doi:10.3762/bjoc.13.211)
Supplement: File 1 — Experimental, instruments section, SEM images, data of TGA, FTIR and BET surface area, virial analysis of the adsorption data for CO2 and NMR spectra. [file Beilstein_J_Org_Chem-13-2131-s001.pdf]

Supporting Information

for

Novel approach to hydroxy-group-containing porous organic polymers from bisphenol A

Tao Wang,[1,2] Yan-Chao Zhao,[1] Li-Min Zhang,[1] Yi Cui,[1] Chang-Shan Zhang,[2] Bao-Hang Han*,[1]

Address: [1]CAS Key Laboratory of Nanosystem and Hierarchical Fabrication, CAS Center for Excellence in Nanoscience, National Center for Nanoscience and Technology, Beijing 100190, China and [2]School of Chemical Engineering, Nanjing University of Science and Technology, Nanjing 210094, China

Email: Bao-Hang Han* - hanbh@nanoctr.cn

*Corresponding author

Experimental, instruments section, SEM images, data of TGA, FTIR and BET surface area, virial analysis of the adsorption data for CO_2 and NMR spectra

Table of contents

Materials and methods..S3

Instrumental characterization……………………………………………………………S3

TGA spectrum...S5

FTIR spectra...S7

NMR spectra...S8

SEM images..S9

BET surface area data..S10

Virial analysis of the adsorption data for CO_2..S12

Materials and methods

Anhydrous potassium carbonate, anhydrous sodium sulfate, 1,3,5-tribromobenzene, 4-bromobenzaldehyde, bisphenol A, 4-formylphenylboronic acid, terephthalic aldehyde (**M1**), *o*-dichlorobenzene, and *p*-toluenesulfonic acid were purchased from Beijing Chemical Reagent Company. Terephthalic aldehyde was purified by recrystallization from ethanol. Ethyl acetate, petroleum ether, dichloromethane, acetone, tetrahydrofuran, and other chemical reagents were used as received. 4,4'-Biphenyldicarboxaldehyde (**M2**) and 1,3,5-tri(4-formylphenyl)benzene (**M3**) were prepared according to previous report [S1]. All condensation reactions to produce **PPOPs** were operated using standard Schlenk line technique.

Instrumental characterization

^{1}H nuclear magnetic resonance (NMR) spectra were recorded on a DMX400 NMR spectrometer (Bruker, Germany) with tetramethylsilane as an internal reference. Solid-state ^{13}C cross-polarization/magic-angle-spinning (CP/MAS) NMR measurements were performed on an Avance III 400 spectrometer ((Bruker, Germany)). Thermogravimetric analysis (TGA) was performed on a Pyris Diamond thermogravimetric/differential thermal analyzer (PerkinElmer Instruments Co. Ltd., USA) by heating the samples at 10 °C min^{-1} to 800 °C in the atmosphere of nitrogen. Infrared (IR) spectra were recorded as KBr pellets using a Spectrum One Fourier transform infrared (FTIR) spectrometer (PerkinElmer Instruments Co. Ltd., USA). The sample was prepared by dispersing the polymers in KBr and compressing the mixtures

to form pellets, and 15 scans were signal-averaged. Field-emission scanning electron microscopy (SEM) observations were performed on an S-4800 microscope (Hitachi, Ltd., Japan) operating at an accelerating voltage of 6.0 kV. The SEM samples were prepared by dropping an ethanol suspension of **PPOP-1**–**PPOP-3** on a silicon wafer and then air drying. Nitrogen adsorption–desorption and hydrogen adsorption experimentations were conducted using an ASAP 2020 M+C accelerated surface area and porosity analyzer (Micromeritics, USA) at 77 K. Carbon dioxide and methane uptake experimentation were performed by using a TriStar II 3020 accelerated surface area and porosity analyzer (Micromeritics, USA). Before measurement, the samples were degassed in vacuo at 120 °C for more than 12 h. A sample of ca. 80.0 mg was used for the gas sorption measurements. The specific surface area result was calculated from nitrogen adsorption data by BET analysis in the relative pressure (P/P_0) range from 0.01 to 0.10 (Supporting Information), whereas pore size and pore size distribution (PSD) were estimated through the original density function theory (DFT). Total pore volume was calculated from nitrogen adsorption–desorption isotherms at $P/P_0 = 0.95$, whereas micropore volume was calculated from nitrogen adsorption isotherm using the *t*-plot method.

Reference

S1. Wang, T.; Zhao, Y.-C.; Luo, M.; Zhang, L.-M.; Cui, Y.; Zhang, C.-S.; Han, B.-H. *Polymer* **2015**, *60*, 26–31. doi: 10.1016/j.polymer.2014.12.072

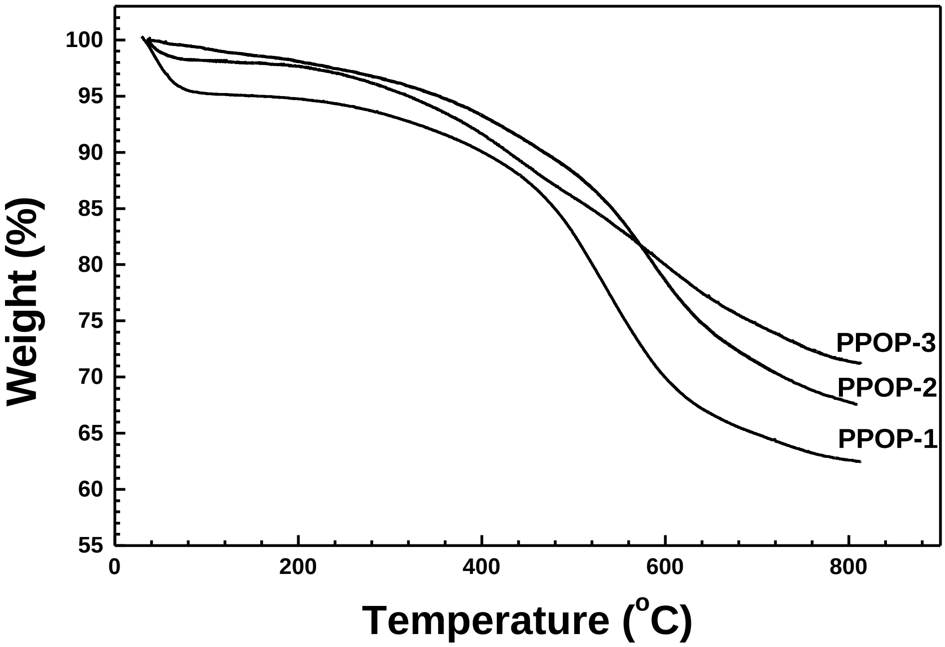

Figure S1: Thermogravimetric analysis (TGA) of **PPOP-1–PPOP-3**.

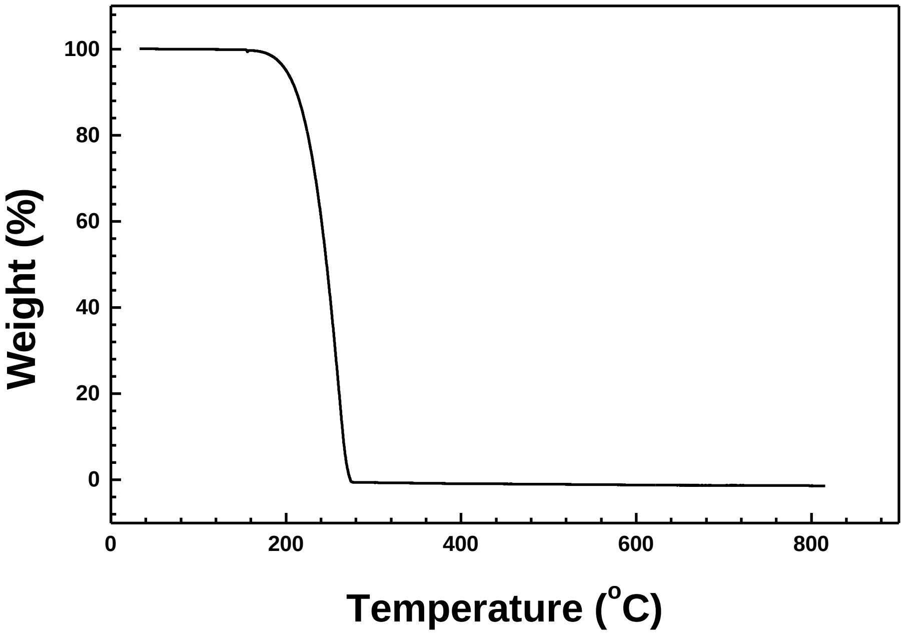

Figure S2: Thermogravimetric analysis (TGA) of BPA.

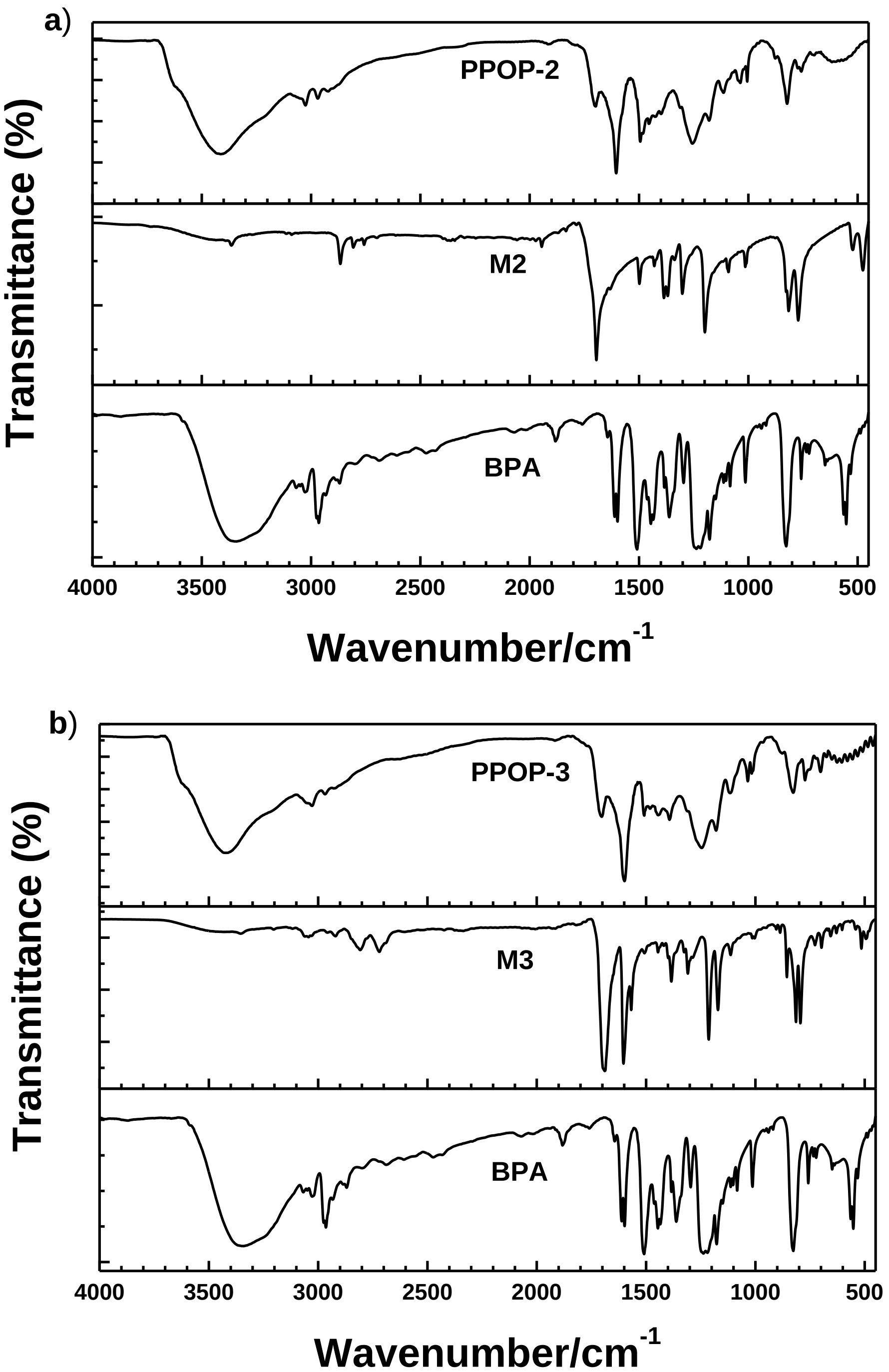

Figure S3: FTIR spectra of (a) 4,4'-biphenyldicarboxaldehyde (**M2)**, **BPA**, and **PPOP-2**; (b) 1,3,5-tris(4-formylphenyl)benzene (**M3**), **BPA**, and **PPOP-3**.

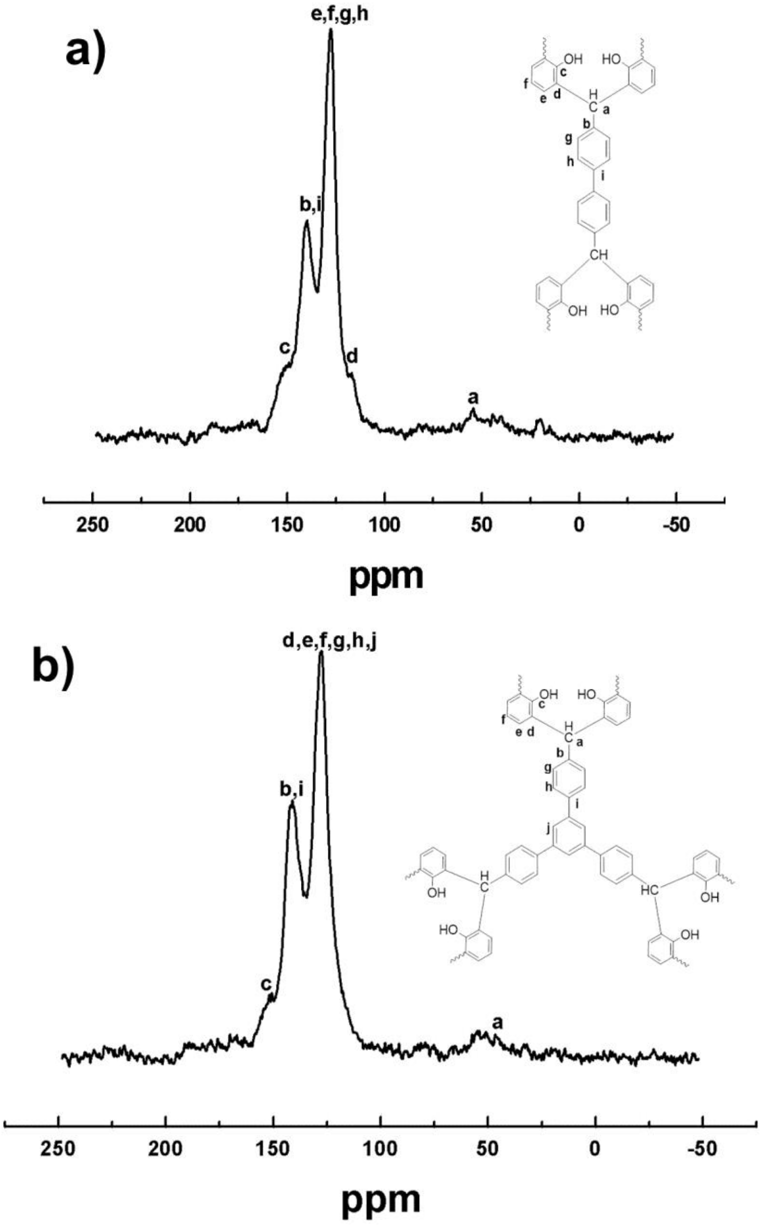

Figure S4: Solid-state ^{13}C CP/MAS NMR spectrum of (a) **PPOP-2** and (b) **PPOP-3** recorded at the MAS rate of 5 kHz.

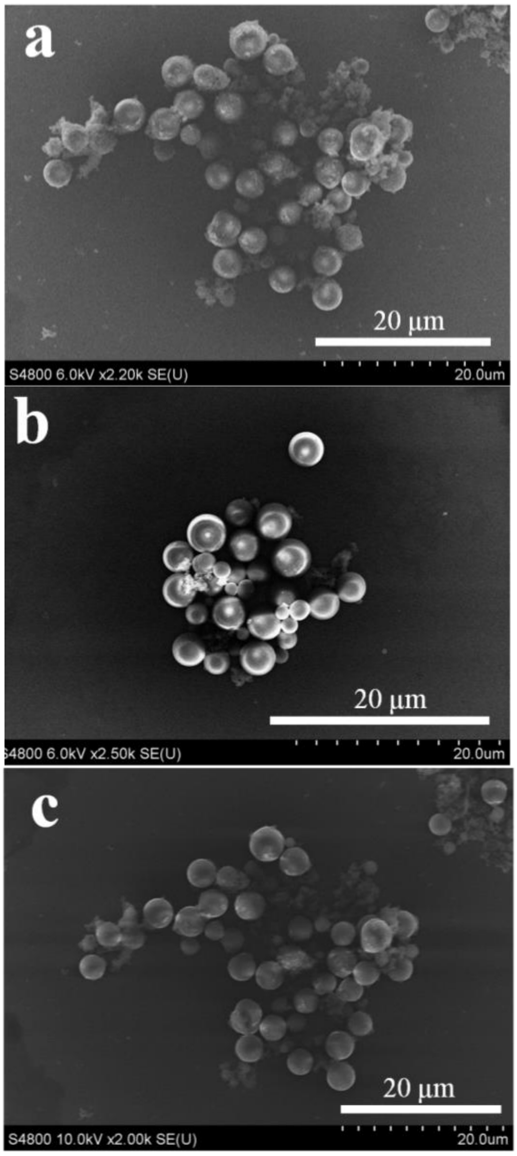

Figure S5: SEM images of (a) **PPOP-1**, (b) **PPOP-2**, and (c) **PPOP-3**.

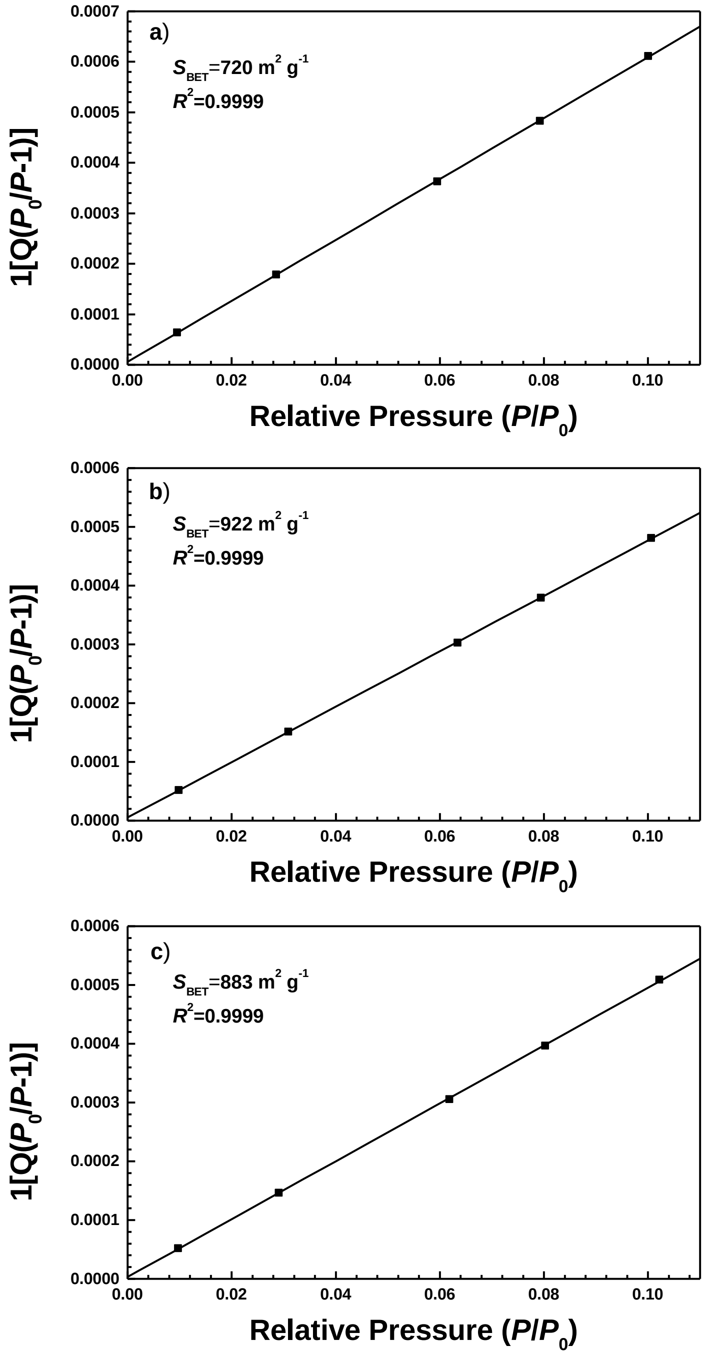

Figure S6: BET specific surface area plots for (a) **PPOP-1**, (b) **PPOP-2**, and (c) **PPOP-3** calculated in the relative pressure range P/P_0 = 0.01–0.10 for the microporous materials, respectively.

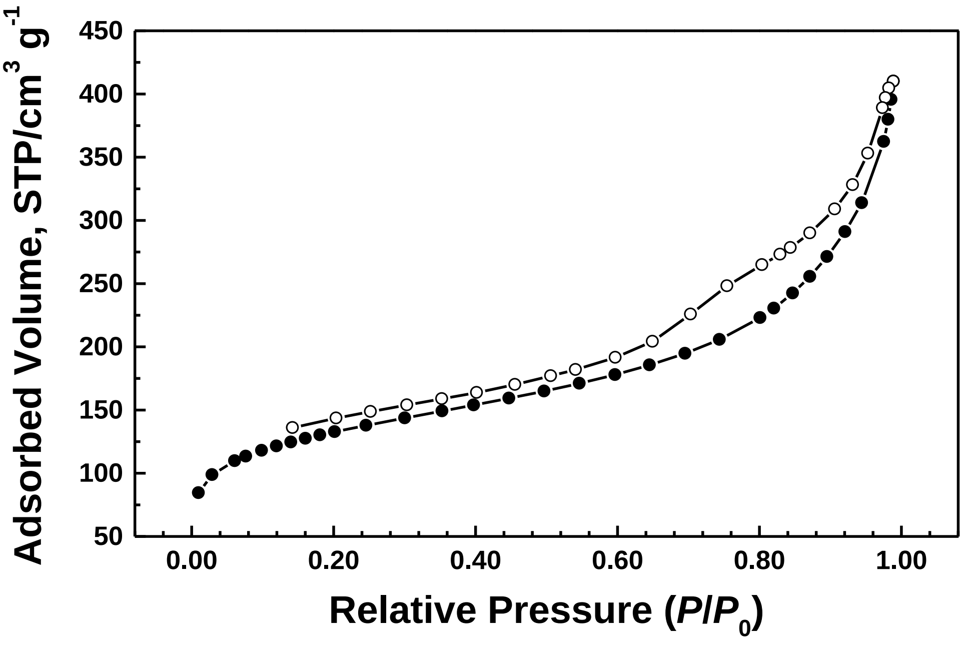

Figure S7: Nitrogen adsorption–desorption isotherms of porous material using phenol as a substitution of BPA to react with **M1** under the same condition.

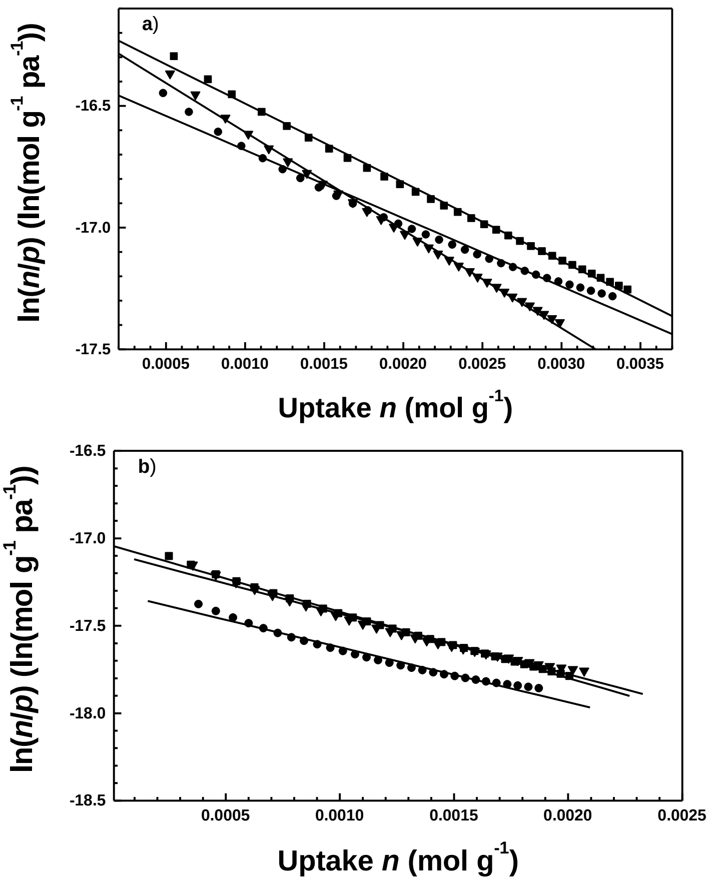

Figure S8: Virial analysis of the adsorption data for CO_2 on **PPOP-1** (downtriangle), **PPOP-2** (circle), and **PPOP-3** (square) at (a) 273 K and (b) 298 K at low pressure range.
